# Causal effect of gut microbiota and diabetic nephropathy: a Mendelian randomization study

**DOI:** 10.1186/s13098-024-01327-7

**Published:** 2024-04-24

**Authors:** Ganyuan He, Jiayi Chen, Wenke Hao, Wenxue Hu

**Affiliations:** Department of Nephrology, Guangdong Provincial People’s Hospital (Guangdong Academy of Medical Sciences), Guangdong Provincial Geriatrics Institute, Southern Medical University, Guangzhou, China

**Keywords:** Causality, Gut microbiota, Diabetic nephropathy, Mendelian randomization

## Abstract

**Background:**

The interaction of dysbiosis of gut microbiota (GM) with diabetic nephropathy (DN) drew our attention and a better understanding of GM on DN might provide potential therapeutic approaches. However, the exact causal effect of GM on DN remains unknown.

**Methods:**

We applied two-sample Mendelian Randomization (MR) analysis, including inverse variance weighted (IVW), MR-Egger methods, etc., to screen the significant bacterial taxa based on the GWAS data. Sensitivity analysis was conducted to assess the robustness of MR results. To identify the most critical factor on DN, Mendelian randomization-Bayesian model averaging (MR-BMA) method was utilized. Then, whether the reverse causality existed was verified by reverse MR analysis. Finally, transcriptome MR analysis was performed to investigate the possible mechanism of GM on DN.

**Results:**

At locus-wide significance levels, the results of IVW suggested that order Bacteroidales (odds ratio (OR) = 1.412, 95% confidence interval (CI): 1.025–1.945, *P* = 0.035), genus Akkermansia (OR = 1.449, 95% CI: 1.120–1.875, *P* = 0.005), genus Coprococcus 1 (OR = 1.328, 95% CI: 1.066–1.793, *P* = 0.015), genus Marvinbryantia (OR = 1.353, 95% CI: 1.037–1.777, *P* = 0.030) and genus Parasutterella (OR = 1.276, 95% CI: 1.022–1.593, *P* = 0.032) were risk factors for DN. Reversely, genus Eubacterium ventriosum (OR = 0.756, 95% CI: 0.594–0.963, *P* = 0.023), genus Ruminococcus gauvreauii (OR = 0.663, 95% CI: 0.506–0.870, *P* = 0.003) and genus Erysipelotrichaceae (UCG003) (OR = 0.801, 95% CI: 0.644–0.997, *P* = 0.047) were negatively associated with the risk of DN. Among these taxa, genus Ruminococcus gauvreauii played a crucial role in DN. No significant heterogeneity or pleiotropy in the MR result was found. Mapped genes (FDR < 0.05) related to GM had causal effects on DN, while FCGR2B and VNN2 might be potential therapeutic targets.

**Conclusions:**

This work provided new evidence for the causal effect of GM on DN occurrence and potential biomarkers for DN. The significant bacterial taxa in our study provided new insights for the ‘gut-kidney’ axis, as well as unconventional prevention and treatment strategies for DN.

**Supplementary Information:**

The online version contains supplementary material available at 10.1186/s13098-024-01327-7.

## Background

Diabetes mellitus (DM) is one of the most common and fastest growing chronic diseases worldwide and it is estimated that there will be 642 million people with DM in 2040 [1]. Diabetic nephropathy (DN), one of the microvascular complications of DM, occurs in almost 20–40% of these patients [2]. DN is characterized clinically with persistent high urinary albumin-to-creatinine ratio over 30 mg/g and/or a continuous decline in estimated glomerular filtration rate to less than 60 ml/min/1.73 m^2 [3]. Patients with DN generally accompany with increased risk of cardiovascular events and progress to end-stage renal disease, which brings a heavy burden on social and high morbidity [4–6]. Despite the management and therapeutic strategies of DN have been established for decades, the quest for truly effective measures continues.

Gut microbiota (GM) is considered as a pivotal “organ” and participates in health maintenance in our whole life [7]. In recent years, intervening the gut-kidney axis for renal diseases treatment tends to be a new research spotlight [8]. Numerous evidences suggested the intestinal flora disorder existed in patients with DN and contributed to DN progression, while probiotics improved DN [9–13]. Imbalance of GM involves in DN progression via GM-derived metabolites, which mainly consist of short-chain fatty acids (SCFAs), bile acids, tryptophan and uremic toxins [14]. Li et al. found the anti-inflammation and anti-fibrosis capability of SCFAs binding with G protein-coupled receptors (GPR)43 or GPR109A in DN mice [15]. Enrichment of SCFAs-producing bacteria may protect against DN. However, another study showed dysbiosis of GM-regulated GPR43 activation aggravated albuminuria in DN through podocyte insulin resistance [16]. These contradictory evidences make confusion and the quality of the evidence of traditional epidemiological studies is concerned due to the limitation of confounding factors or reverse causality. Whether there is a causal relationship between GM and DN is still unclear. Accordingly, it is necessary to figure out the causal connection between GM and DN at the genetic level.

Mendelian randomization (MR) analysis is a study method to explore the causal effect of exposures and outcomes by using genetic variations as instrumental variables (IVs) [17]. Naturally, genetic variation is inherited randomly and the DNA phenotype is dependent on parent, thus the causality between exposures and outcomes cannot be influenced by multifarious confounding factors [18]. Similarly, the outcome does not change the intrinsic genetic variations and thus avoids reverse causation. Compared to the traditional observational studies, MR analysis provides a possibility to study the causal effect between exposures and outcomes with mitigating the bias from confounding factors and reverse causation [19]. 

To identify the link between GM and DN risk, we conducted a two-sample MR method using genome-wide association study (GWAS) summary data. The findings of the study might provide new insights into the mechanism of GM on DN, detection and the potential therapeutic target.

## Methods

### Study design

**Fig. 1:**
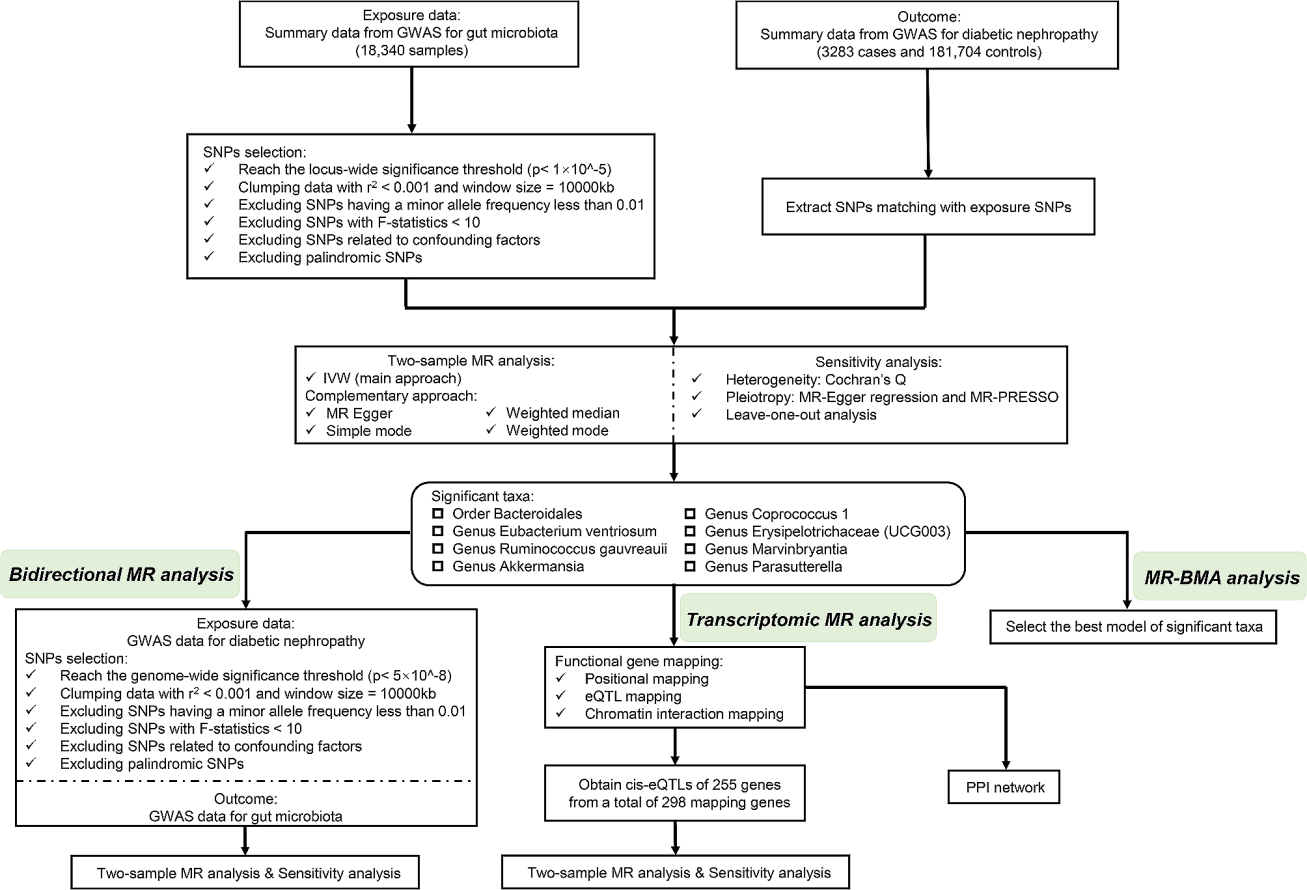
Flowchart illustrating the present MR study. GWAS, genome-wide association study; SNPs, single nucleotide polymorphisms; IVW, inverse variance weighted; MR-PRESSO, Mendelian Randomization pleiotropy residual sum and outlier; MR-BMA, Mendelian randomization-Bayesian model averaging; eQTL, expression quantitative trait loci; PPI, protein-protein interactions

**Fig. 2:**
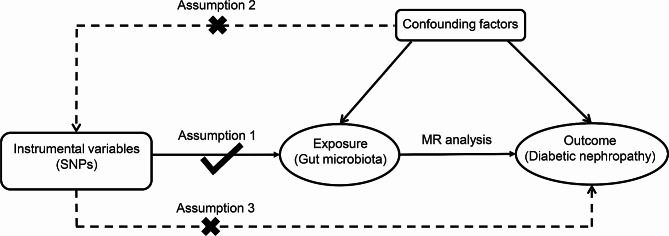
Three core assumptions of MR analysis

In this work, two-sample Mendelian Randomization was used to assess the association between GM and DN risk. Then, transcriptome Mendelian Randomization was conducted to further explore the mechanism of specific bacterial taxa on DN. The flowchart of the study is shown in Fig. 1. Three core assumptions of standard MR were complied to make the MR results convincing: [1] the selected IVs must be significantly associated with GM taxa; [2] the IVs included in MR analysis did not correlate with the confounders that affected both GM and DN; [3] there was no other connection between IVs and DN, except for the influence of GM (Fig. 2) [20]. 

### Data sources of GM and DN

The GWAS summary statistics of GM were available from the MibioGen research [21]. These data were derived from the largest GWAS meta-analysis which involved 18,340 participants from 24 cohorts [22]. Five levels (phylum, class, order, family, genus) and 211 bacterial taxa in total were identified while 15 unknown bacterial taxa were excluded in the study. DN GWAS summary data comprising 3283 cases and 181,704 controls were obtained from FinnGen Release 5, published in 2021 [23]. The inclusion criterion of DN patients was based on ICD-10 code N083.

### Instrumental variables selection

The IVs were chosen based on the following criteria: [1] The number of single nucleotide polymorphisms (SNPs) was too small when the candidate SNPs were filtered with the genome-wide significance threshold (*p* < 5 × 10^-8) and thus might result in missing potential findings. In this study, locus-wide significance threshold (*p* < 1 × 10 − 5) was used to select the potential SNPs associated with GM; [2, 24, 25] To avoid linkage disequilibrium, the SNPs were only retained after the clumping process (r^2^ < 0.001 and window size = 10,000 kb); [3] Proxy SNPs with linkage disequilibrium R^2^ > 0.8 were found to substitute the selected SNPs, which were not matched in GWAS of DN; [4] the SNPs were removed with a minor allele frequency (MAF) less than 0.01; [5] The strength of each SNP was measured by the F-statistics which was calculated by the following formula: $$ F={R}^{2}(N-2)/(1-{R}^{2})$$, R^2^ was the proportion of the variability of bacterial taxa explained by each SNP and N was the sample size [26]. For eliminating weak IVs, only those SNPs with F-statistics greater than 10 were kept; [6, 27] To avoid the confounders related to SNPs affected DN, PhenoScanner V2, a database of human genotype-phenotype associations was used to recognize and weed out those SNPs linked to the confounding factors (hypertension, autoimmune disease, etc.); [7, 28] Finally, the palindromic SNPs were eliminated to assure the effects of SNPs on GM corresponded to the consistent allele as the effects on DN.

### MR analysis

In this work, a series of approaches containing inverse variance weighted (IVW), MR-Egger, weighted median, simple model and weighted model were carried out to clarify whether there was a causality between GM and DN. Among these methods, IVW was the primary method to determine the effect of GM on DN and the results were reliable without horizontal pleiotropy [29]. In addition to IVW, other methods were utilized to acquire robust causal relationship. Firstly, the results of MR-Egger were in coincidence with IVW when there was no pleiotropy [30]. Weighted median could still assess the causality accurately even if the number of the invalid SNPs exceeded 50% [31]. Simple model and weighted model exhibited less bias and lower type I error rates than other methods mentioned above but smaller power to detect the causal effect than IVW and weighted median approaches [32]. We regarded there was a causal relationship between GM and DN if IVW_*p*−value_ was less than 0.05 and supposed that the MR results would be more convinced if one or more other additional methods reached statistical significance. The following formula was employed to calculate the OR: $$ OR{= e}^{beta}$$, while beta represented the effect size of the exposure on the outcome. Meanwhile, the 95%CI was calculated by the following formula: $$ beta\pm 1.96\times SE$$ [29]. 

### Sensitivity analysis

Since the IVs concluded in MR analysis were derived from a variety of cohorts study, sensitivity tests should be done to examine the robustness of MR results. To prevent heterogeneity effect on the causality, Cochran’Q tests were performed to discover the heterogeneity among selected SNPs. Cochran’Q tests_*p*−value_ > 0.05 indicated the absence of heterogeneity effect. IVW random effects model was applied when significant heterogeneity existed.

For ensuring that the causal effect was not confounded by genetic variations affecting the outcome through pathways other than the exposure of interest, MR-Egger regression was utilized to detect the potential pleiotropy bias. If significant horizontal pleiotropy existed (p-value < 0.05), the result was susceptible attributing to violation of the MR assumptions [30]. Besides, to enhance the precision of causal effect estimates, Mendelian Randomization pleiotropy residual sum and outlier (MR-PRESSO) was indispensable for identifying and correcting for pleiotropic outliers [33]. Finally, we also applied the leave-one-out analysis which iteratively excluded each SNP to identify the influential variants and the robustness of the results was affirmed given the minimal fluctuations in the overall confidence intervals upon the sequential exclusion of SNPs [34]. 

### Mendelian randomization-bayesian model averaging (MR-BMA)

After standard MR analysis finished, several bacterial taxa with plenty of common genetic variants were discovered to be significantly correlated with DN risk. Mendelian randomization-Bayesian model averaging (MR-BMA), a multivariable MR approach, is able to identify causal risk factors from a high-throughput experiment and determine which are the primary causal contributors of disease risk within a cluster of correlated risk factors that share common genetic predictors [35]. Therefore, to correct the effect of “measured polymorphism” and find the specific taxon that was the most likely to be causal, we used MR-BMA to analyze those significant taxa with IVW_*p*−value_ <0.05 in previous MR analysis. Posterior probability (PP) was calculated for all specific models (i.e., one exposure or a combination of multiple exposures) and the best model was selected based on the PP value. The marginal inclusion probability (MIP) for each risk factor, was derived from the sum of the PPs across all models in which the respective risk factor was incorporated. It was used to rank the causal relationship of the exposures with the outcome. Besides, we also calculated model-averaged causal effects (MACE) which demonstrated the direct causal effect of a risk factor on the outcome by averaging the effects across all pertinent models. In the MR-BMA analysis, outlier and influential instruments were recognized by Q-statistics and Cook’s distance, respectively. SNPs were eliminated under the condition that Q-statistic value over 10 or Cook’s distance exceeded the threshold.

### Reverse MR analysis

To clarify whether the alteration of GM was impacted by DN, we performed reverse Mendelian Randomization analysis to test the causal effect of DN on significant bacterial taxa with IVW_*p*−value_ <0.05 in standard MR analysis. The SNPs selection criteria were shown in Fig. 1. The process of reverse MR was the same as MR we described previously.

### Transcriptomic MR analysis

In order to explore the role of GM in the DN pathogenesis, we further performed the transcriptome Mendelian Randomization analysis on significant taxa and DN. For each taxon that was significant in MR analysis, we entered GWAS summary statistics and all selected SNPs as ‘pre-defined lead SNPs’ in SNP2GENE function of FUMA GWAS, a platform that can be used to annotate, prioritize, visualize and interpret GWAS results [36]. These SNPs were mapped to gene by positional, expression quantitative trait loci (eQTLs) and chromatin interaction mapping approaches. Then, to comprehend how genes interacted at protein level, STRING (Version 12.0) was used to create the protein-protein interactions (PPI) network with medium confidence (0.4) as recommended minimum interaction score while Cytoscape (V3.10.1) was used to analyze the PPI data [37, 38]. To validate the causal association of mapped gene and DN, we acquired the cis-eQTLs of each mapped gene from the eQTLGen consortium, which incorporated 37 repositories with a total of 31,684 blood samples and cis-eQTLs (SNP gene distance < 1 Mb, FDR < 0.05) for 16,987 genes, and the majority of individuals were of European ancestry. The significant cis-eQTLs with false discovery rate (FDR) < 0.05 and the allele frequencies were obtained in cis-eQTLs of eQTLGen phase I [39]. For deeper MR analysis, standard error and beta (the SNP effect on trait) were calculated by the following formula respectively: $beta=\frac{\text{z}}{\sqrt{{2p(1-p)(n+z}^{2})}}$ and $SE=\frac{1}{\sqrt{{2p(1-p)(n+z}^{2})}}$ [40]. The application of an extremely low correlation standard might cause the omission of causative variants; hence, we screened linkage disequilibrium clumped (r^2^ < 0.1) cis-eSNPs as IVs [41]. The SNPs selection criteria were shown in Fig. 1. The MR analysis and sensitivity analysis remained consistent with previous methods, and we accounted for multiple testing issues by applying an FDR correction. The result showing an FDR < 0.05 was treated as significant [42]. 

### Statistical analysis

All the MR analyses were conducted by “TwoSampleMR” and “MR-PRESSO” packages in R version 4.3.1. PPI data and graph display were performed in Cytoscape V3.10.1.

## Results

### Causal effects and sensitivity analysis of gut microbiota on DN

**Fig. 3:**
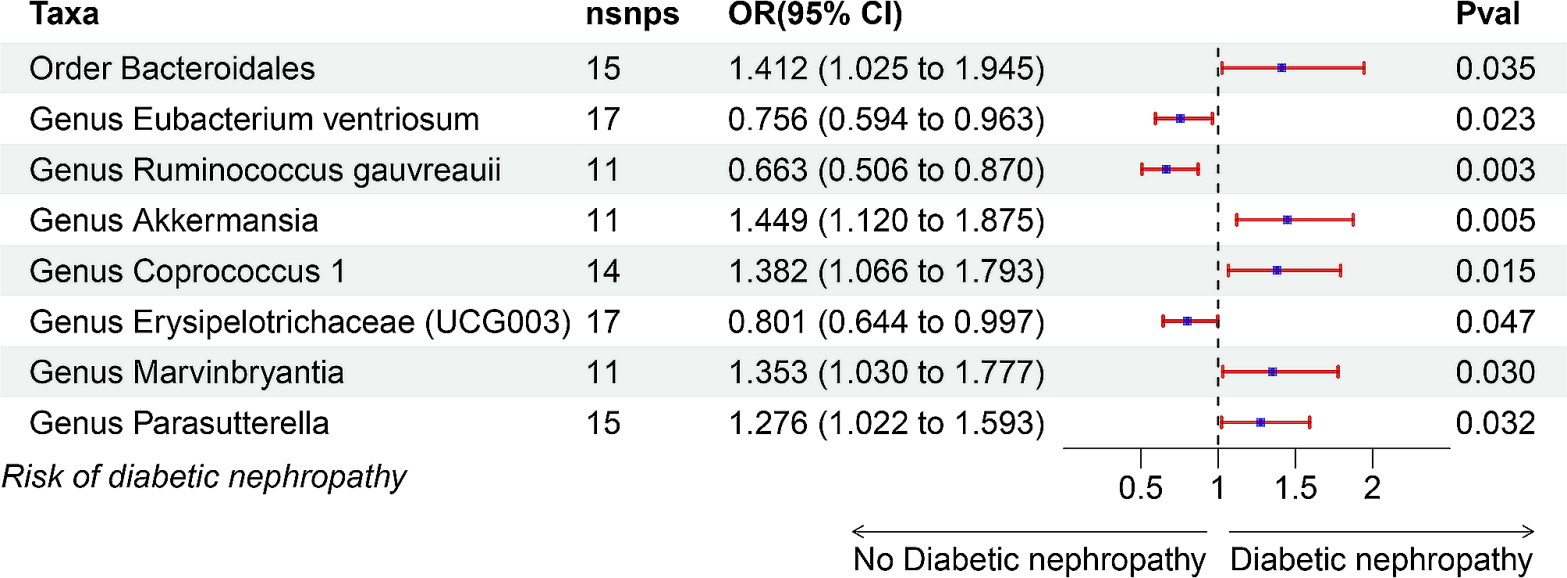
Forest plot of IVW results for causal effect of GM on risk of DN

In the study, 8 bacteria taxa were found to be significantly associated with risk of DN after using MR analysis for a total of 196 taxa (excluding 15 unknown taxa) and the forest plot of causal effect between these taxa and DN was shown in Fig. 3. The results of IVW showed that order Bacteroidales (odds ratio (OR) = 1.412, 95% confidence interval (CI): 1.025–1.945, *P* = 0.035), genus Akkermansia (OR = 1.449, 95% CI: 1.120–1.875, *P* = 0.005), genus Coprococcus 1 (OR = 1.328, 95% CI: 1.066–1.793, *P* = 0.015), genus Marvinbryantia (OR = 1.353, 95% CI: 1.037–1.777, *P* = 0.030) and genus Parasutterella (OR = 1.276, 95% CI: 1.022–1.593, *P* = 0.032) were positively correlated with risk of DN. Conversely, higher abundance of genus Eubacterium ventriosum (OR = 0.756, 95% CI: 0.594–0.963, *P* = 0.023), genus Ruminococcus gauvreauii (OR = 0.663, 95% CI: 0.506–0.870, *P* = 0.003) and genus Erysipelotrichaceae (UCG003) (OR = 0.801, 95% CI: 0.644–0.997, *P* = 0.047) were associated with lower risk of DN. The MR estimates of weighted median indicated that order Bacteroidales (OR = 1.594, 95% CI: 1.086–2.341, *P* = 0.017) and genus Coprococcus 1 (OR = 1.550, 95% CI: 1.099–2.184, *P* = 0.012) were positively correlated with DN while a negative link between genus Ruminococcus gauvreauii (OR = 0.645, 95% CI: 0.445–0.993, *P* = 0.020) and DN (Table S1). The details of instrumental variations of each significant taxon were shown in Table S2.

In sensitivity analysis, no heterogeneity was found in the selected IVs of any significant bacterial taxa based on Cochrane’s Q tests. The findings from the MR-Egger regression and MR-PRESSO global test suggested that there was no evidence for horizontal pleiotropy to misrepresent the causality of the gut microbiota on DN (Table 1). Eventually, leave-one-out analysis suggested no influential SNP dominating the casual relationship of GM and DN (Figure S1).

**Table 1:** Heterogeneity and pleiotropy test results of genetic variants

Gut microbiota	Heterogeneity				Pleiotropy		MR-PRESSO	
	MR Egger		IVW		MR Egger regression		Global Test	
	Cochran’s Q	P-value	Cochran’s Q	*P*-value	Egger intercept	*P*-value	RSSobs	*P*-value
Order Bacteroidales	15.356	0.167	15.497	0.215	0.009	0.756	17.487	0.271
Genus Eubacterium ventriosum	4.886	0.978	4.928	0.987	-0.008	0.840	5.532	0.992
Genus Ruminococcus gauvreauii	7.149	0.521	8.626	0.473	0.049	0.259	10.636	0.517
Genus Akkermansia	9.237	0.323	9.267	0.413	0.006	0.876	11.398	0.471
Genus Coprococcus 1	4.362	0.930	5.285	0.917	-0.024	0.359	6.226	0.935
Genus Erysipelotrichaceae (UCG003)	8.428	0.866	8.476	0.903	0.006	0.829	9.624	0.933
Genus Marvinbryantia	4.919	0.766	5.135	0.822	-0.022	0.655	6.219	0.846
Genus Parasutterella	4.045	0.969	6.514	0.888	0.061	0.144	7.858	0.893

IVW, inverse variance weighted; MR-PRESSO, Mendelian Randomization pleiotropy residual sum and outlier

### MR-BMA analysis of gut microbiota on DN

We included the order Bacteroidales, genus Eubacterium ventriosum, genus Ruminococcus gauvreauii, genus Akkermansia, genus Coprococcus 1, genus Erysipelotrichaceae (UCG003), genus Marvinbryantia and genus Parasutterella which had significant causal relationship with DN in MR-BMA analysis. During running process of MR-BMA method, no invalid and influential instrument was detected. Posterior probability was calculated for each model and model-specific causal estimates were shown in Table 2. Genus Ruminococcus gauvreauii was the best model with the highest score of PP (0.309) among the top 10 models and the model-specific casual estimates was − 0.369. Bacterial taxa were prioritized based on their significance, as determined by the MIP (Table 3). Consequently, the MR-BMA results revealed that genus Ruminococcus gauvreauii was the crucial causal risk factor and might reduce the risk of DN with the highest rank from MIP (MIP = 0.935, MACE = -0.374, *p* = 0.002) [35]. 

**Table 2:** Ranking of gut microbiota for diabetic nephropathy according to posterior probability

Rank	Models or sets of risk factor(s)	*PP*	Model-specific causal estimates
1	Ruminococcus gauvreauii	0.309	-0.369
2	Ruminococcus gauvreauii, Marvinbryantia	0.251	-0.449, 0.274
3	Bacteroidales, Ruminococcus gauvreauii, Marvinbryantia	0.102	0.269, -0.403, 0.321
4	Ruminococcus gauvreauii, Coprococcus 1	0.039	-0.442, 0.196
5	Bacteroidales, Ruminococcus gauvreauii	0.034	0.197, -0.325
6	Ruminococcus gauvreauii, Akkermansia	0.024	-0.345, 0.154
7	Bacteroidales	0.018	0.282
8	Ruminococcus gauvreauii, Erysipelotrichaceae (UCG003)	0.017	-0.332, -0.130
9	Ruminococcus gauvreauii, Erysipelotrichaceae (UCG003), Marvinbryantia	0.016	-0.412, -0.135, 0.277
10	Eubacterium ventriosum, Ruminococcus gauvreauii	0.015	-0.123, -0.35

PP, posterior probability.

**Table 3:** Ranking of risk factors for diabetic nephropathy according to marginal inclusion probability

Rank	Gut microbiota	MIP	MACE	*P* value
1	Genus Ruminococcus gauvreauii	0.935	-0.374	0.002
2	Genus Marvinbryantia	0.463	0.131	0.031
3	Order Bacteroidales	0.232	0.061	0.127
4	Genus Coprococcus1	0.096	0.018	0.706
5	Genus Akkermansia	0.078	0.013	0.797
6	Genus Erysipelotrichaceae (UCG003)	0.064	-0.009	0.873
7	Genus Eubacterium ventriosum	0.05	-0.006	0.933
8	Genus Parasutterella	0.027	0.002	0.995

MIP, marginal inclusion probability; MACE, model-averaged causal effect

### Reverse causation of DN on significant gut microbiota

To investigate whether genetically predicted DN was a causal risk factor for GM dysbiosis, reverse Mendelian Randomization was applied to the eight significant bacterial taxa. The estimates of MR-Egger presented that genus Parasutterella was significantly associated with DN (*p* = 0.026), as shown in Table S3. Nevertheless, the MR results brought concerns accounting for horizontal pleiotropy proved by MR-Egger regression analysis (Egger intercept = -0.063, *p* = 0.018). Additionally, the sensitivity analysis of both of genus Marvinbryantia and genus Parasutterella showed the evidence of heterogeneity (see in Table S4). For fortifying the robustness of MR results, IVW-random effect method was employed and the results revealed no significant link among DN and these two taxa. Thus, we considered the reverse causal relationships between DN and these taxa did not exist.

### Gene mapping by the selected SNPs and PPI network

**Fig. 4:**
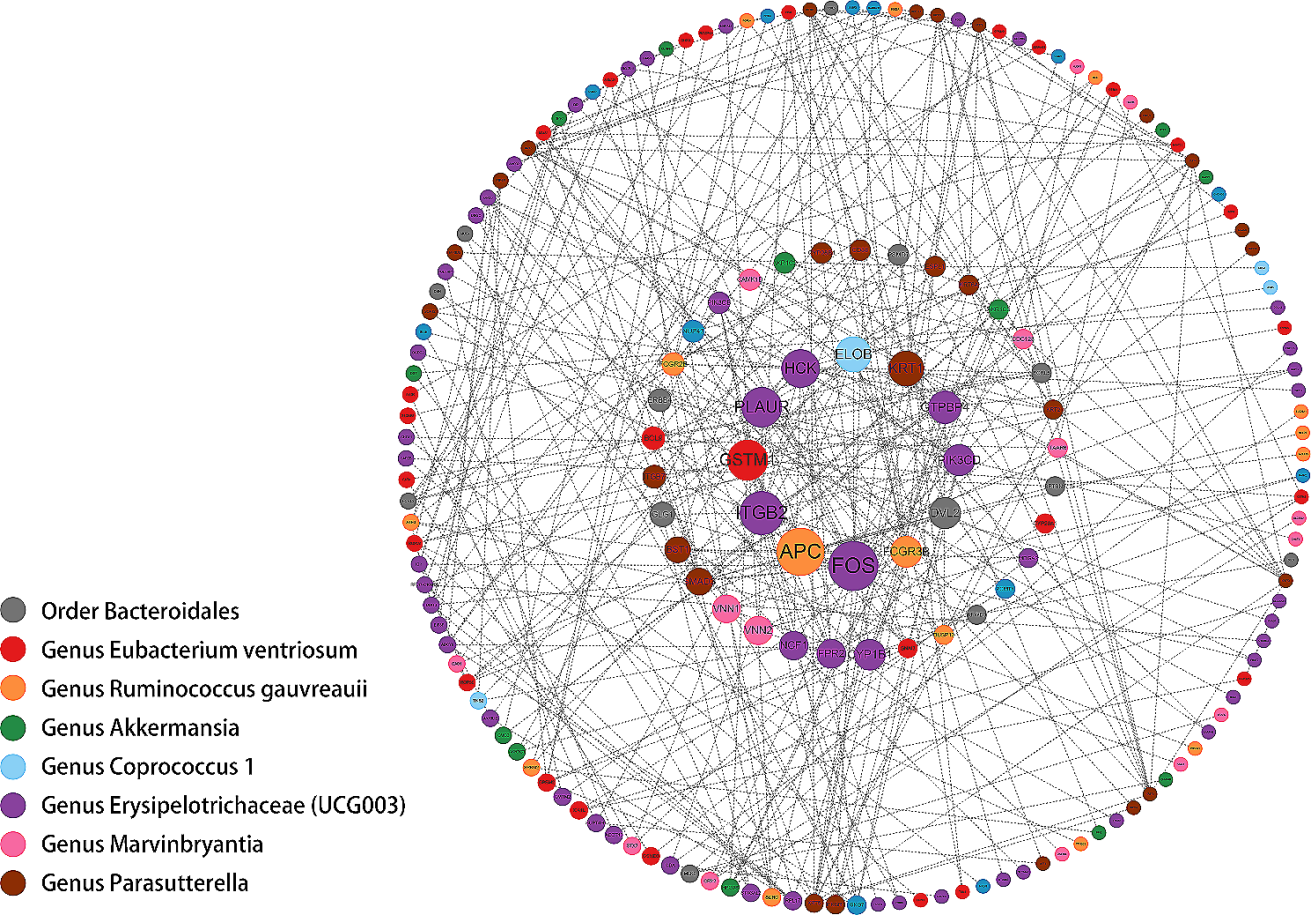
PPI network of significant bacterial taxa related mapped genes. Eight bacterial taxa are classified by distinct colors. The nodes represent the encoded protein and the edges represent the interaction among them. Betweenness centrality score is reflected by the size of node and font size, that larger size accompanied with greater BC

For a better understanding of the biological relevance of previous findings, appraisal of the functional annotations of the genetic variants regarded as IVs in prior MR analysis was carried out in FUMAGWAS tool. 298 genes were mapped from chosen abundant SNPs through three different annotation methods including positional, eQTL and chromatin interaction mapping. All mapped gene were displayed in Table S5. Afterwards, the potential proteins were searched according to the mapped genes in STRING. We used Cytoscape software to analyze PPI data and visualize the PPI network as shown in Fig. 4. In the constructed protein network, which encompassed 177 nodes and 355 edges, the magnitude of each node’s circle represented its betweenness centrality (BC) parameter. Notably, FOS, APC, ITGB2, GSTM1, and PLAUR were distinguished as the top five of proteins exhibiting the highest BC values within the network.

### Transcriptomic MR analysis of mapped gene of DN

**Fig. 5:**
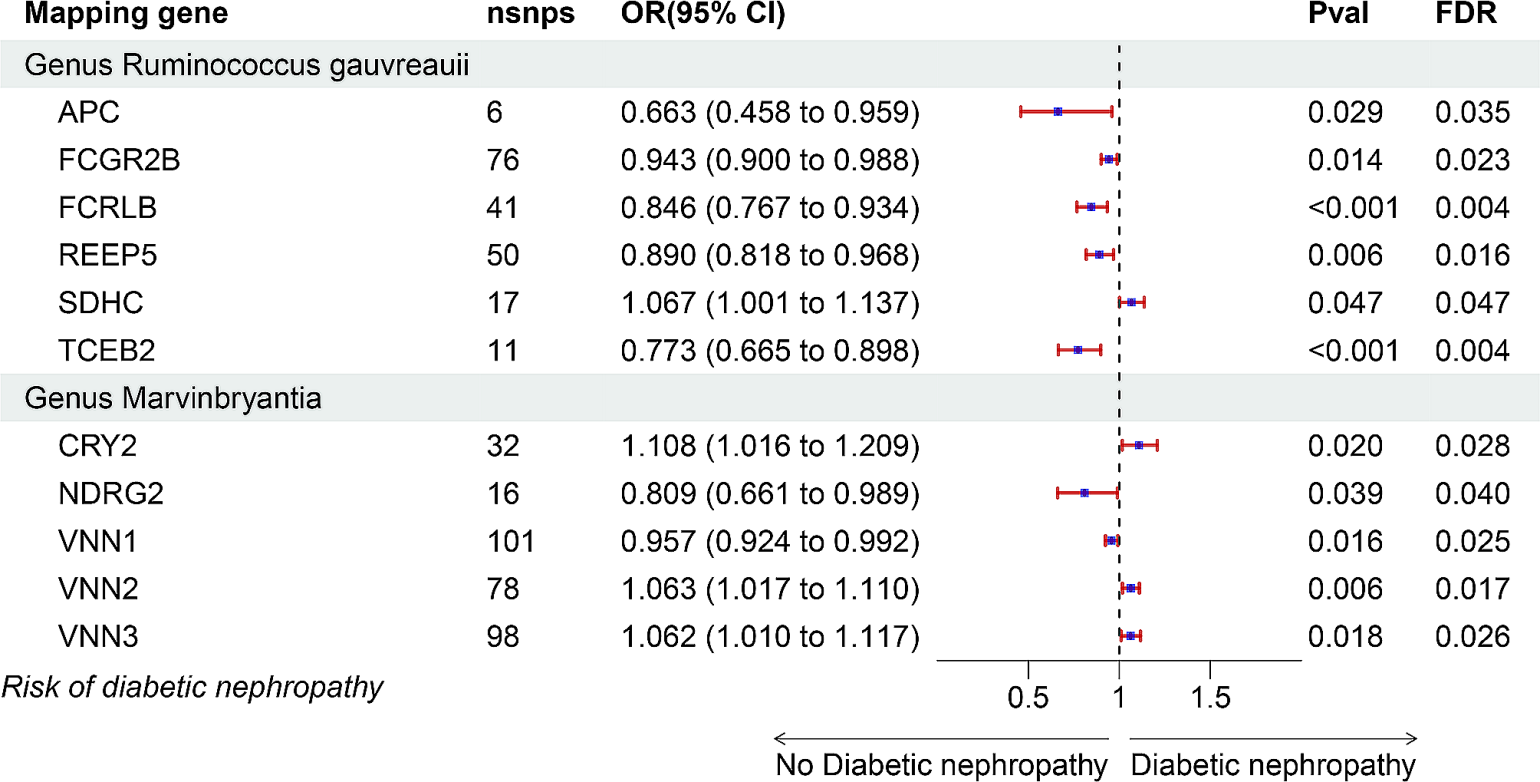
Forest plot of causality between mapped genes and DN

As illustrated in methods, we gained cis-eQTLs of mapped gene from eQTLGen consortium and explore the causal effect of cis-eQTLs on DN risk via MR analysis. A total of 48 mapped gene expression were significantly associated with risk of DN (FDR < 0.05). Among these gene, APC (OR = 0.663, 95% CI: 0.458–0.959, FDR = 0.035), FCGR2B (OR = 0.943, 95% CI: 0.900-0.988, FDR = 0.023), FCRLB (OR = 0.846, 95% CI: 0.767–0.934, FDR = 0.004), REEP5 (OR = 0.890, 95% CI: 0.818–0.968, FDR = 0.016) and TCEB2 (OR = 0.773, 95% CI: 0.665–0.898, FDR = 0.004) were negatively correlated with DN risk in genus Ruminococcus gauvreauii while high expression SDHC (OR = 1.067, 95% CI: 1.001–1.137, FDR = 0.047) prompted DN occurrence (Fig. 5). The complete MR results of other significant genes were shown in Table S6. Then, no heterogeneity was detected among individual eQTLs. Except for Gene GLG1 in the order Bacteroidales and DPEP3 in the genus Erysipelotrichaceae (UCG003), which were excluded owing to presence of pleiotropy, distortion of the influence of other mapped genes on DN was considered low (Table S7). Furthermore, no influential SNP was found in leave-one-out analysis of all mapped genes (Figure S2-9).

## Discussion

High prevalence and morbidity of DN drives us to pursuit the pathogenesis and new therapeutic targets for patients with DN. This study marked the inaugural effort to elucidate the causal effect of GM on DN through a series of MR analysis. The current investigation not only suggested that genus Ruminococcus gauvreauii was predominantly and causally related with a reduced risk of DN via gene FCRG2B, but also revealed another 7 bacterial taxa had causal effects on DN. The findings signified the essential contribution of GM to the progression of DN and provided a genetic-level reference for further research.

Previously, a great deal of studies investigated the interaction between gut microbiota and several diseases, like diabetes mellitus, autoimmune disease, chronic kidney disease, etc [43–45]. Alteration of gut microbiome attributed to curative effect and clinical outcomes [45, 46]. Similarly, numerous studies explored the adverse effect of GM dysbiosis on DN and the beta diversity of GM in DN differed from healthy control [11, 47, 48]. In our study, order Bacteroidales, genus Akkermansia, Coprococcus 1, Marvinbryantia and Parasutterella were found to be risk factors for DN while the others were the protective factors. A recent meta-analysis summarized the changes of abundance of gut microbiota based on current reported studies and found that the abundance of genus Akkermansia increased in patients with DN while genus Coprococcus varied from distinct studies [11]. Augmented abundance of genus Akkermansia might be a risk factor of DN which was consistent with our study. However, the actual mechanism of genus Akkermansia affecting DN was still uncovered. Genus Akkermansia was reported to negatively modulate glucose metabolism via interferon-γ and improved insulin sensitivity [49, 50]. Restoral of abundance of genus Akkermansia might prevent DN development via SCFAs producing [48]. Conversely, genus Akkmeransia played a crucial role in gut-immune axis and promoted M1 macrophages polarization which secreted excessive inflammatory factors and aggravated kidney injury [51]. Besides, the abundance of genus Akkmeransia was positively correlated with renal failure biomarkers and increased along with CKD progression, which indicated that genus Akkmeranisa participated in uremic toxin production and led to kidney dysfunction [52–54]. More extensive investigations were warranted to characterize and offer a comprehensive understanding of the contentious effect of genus Akkmeransia on DN. Besides, genus Parasutterella was positively related with inflammatory cytokines like lipopolysaccharide and interleukin (IL)-8 but negatively with IL-10 in type 2 diabetes mellitus (T2DM) mice, while probiotic treatment maintained barrier integrity and ameliorated inflammation through decreasing enrichment of genus Parasutterella [55].

There was no traditional epidemiological study on the relationship of other significant bacterial taxa found in our study and DN. Gut metabolites, insulin resistance, local RAS activation, inflammation and mucosal immunity disorder accounting for GM dysbiosis were the primary mechanisms to DN [14]. Acetate, butyrate and propionate, which were generated by anaerobic fermentation of dietary fibers, were main components of SCFAs. It was known that Bacteroidetes and Firmicutes stood as the predominant phyla in human intestinal microbiota, with Bacteroidetes members were primary source of acetate and propionate, whereas Firmicutes were mainly engaged in butyrate synthesis [56, 57]. Acetate played a pivotal role in dysregulation of cholesterol homeostasis through the activation of GPR43, and consequently contributed to tubulointerstitial injury in DN [58]. This might be the potential mechanism of order Bacteroidales affecting DN. Regarding to butyrate, several molecular pathways took part in DN protection. (i) Butyrate was speculated to improve insulin resistance via GPR43. SCFAs could bind to GPCRs like GPR43 and involved in GPCRs mediated signaling pathways [59]. A previous study demonstrated dysregulation of GPR43 modulated by dysbiosis of GM resulted in podocyte insulin resistance and glomerular injury in DN while another research discovered butyrate reversed insulin resistance through GPR43 mediated suppression of oxidative stress and NF-κB signaling in mice model, which prevented mesangial matrix deposition and renal fibrosis [16, 60]. (ii) Dysbiosis-induced acetate overproduction was implicated in the renal damage observed in early DN through the activation of intrarenal RAS [10]. Lei W and colleagues demonstrated that sodium butyrate ameliorated angiotensin II-induced kidney injury via inhibition of renal (pro)renin receptor and intrarenal RAS [61].

Apart from the previous elaboration of protective effects of butyrate on DN, a few works explored the role of butyrate in immunity regulation [62–64]. Man Y, et al. reported that sodium butyrate was capable to alleviate vacuolar degeneration of renal tubules and tubular epithelial cells exfoliation, via attenuating inflammation activation mediated by PI3K/Akt/NF-κB pathway in high glucose induced human monocyte-macrophages [65, 66]. Butyrate could regulate Treg/Th17 equilibrium by promoting regulatory T cell differentiation while inhibiting Th17 helper T cell [63]. Kathrin Eller, et al. claimed the role of CD4(+)Foxp3(+) Tregs in improving insulin sensitivity and diabetic nephropathy [67]. Oppositely, Th17 cell mediated inflammation and produced IL-17, a proinflammatory cytokine, and hence increased the risk of T1DM due to islet inflammation and β-cells destruction [68, 69]. Thereby, butyrate might engage in prevention from DN by adjusting Treg/Th17 ratio. Both genus Eubacterium ventriosum, Ruminococcus gauvreauii and Erysipelotrichaceae (UCG003) belongs to Firmicutes phylum and all these bacterial taxa mainly produce butyrate. Based on the aforementioned protective effects of butyrate on DN and the results in MR analysis, we hypothesized these taxa might played an essential role in DN improvement mediated by butyrate.

Although genus Coprococcus 1 and Marvinbryantia also produced butyrate, the current studies on interaction among genus Coprococcus 1, diabetes and kidney disease were discrepant and whether genus Coprococcus 1 took part in DN development was still unknown. Further study should be done to figure out the potential mechanism. Additionally, some researchers showed that genus Marvinbryantia was positively correlated with inflammatory cytokines and might induce inflammatory response in high glucose environment [70, 71]. According to our study, we did transcriptome MR analysis and found deleterious effect of VNN2 on DN. VNN 2 was detected high expression in kidney tissue [72]. The protein Gpi80, encoded by the VNN2, exhibited surface aggregation on activated migrating neutrophils and potentially modulated neutrophil adhesion and migration [73]. Thus, we implied that genus Marvinbryantia involved in pathological exacerbation of DN by inflammation activation.

Several GM related genes had been found to have causal relationship with DN in this work. Among these potential genes, FCGR2B relevant to ‘true factor’ genus Ruminococcus gauvreauii might reduce risk of DN. FcγRIIB, a unique inhibitory Fcγ receptor known to be expressed on various immune cells like B cells, macrophages and granulocytes, is the product of gene FCGR2B [74]. FcγRIIB has been widely studied in autoimmune-mediated kidney diseases [75–77]. Acute kidney injury aggravated lupus activity through spleen tyrosine kinase (Syk)/neutrophil extracellular traps pathways in FcγRIIB deficient mice [75]. A recent study on lupus nephritis revealed that FcγRIIB conducted inhibitory effect on IL-1β production, which was elevated in several nephritis, in kidney macrophages through Syk signaling pathways [77]. Furthermore, FcγRIIB limited adaptive immunity by inducing CD8 + T cell apoptosis while suppressing CD8 + T cell response mitigated renal injury and fibrosis [78, 79]. Therefore, we inferred the influence of genus Ruminococcus gauvreauii on DN through FCGR2B. Regardless of other genes found causation with DN in MR analysis had not been reported to be related to DN, it might provide new insights for future investigations into the mechanism underlying the interaction between GM and DN.

Although mature management and treatment on DN have been established in these years like blood glucose monitoring and application of RAS inhibitors or sodium-glucose cotransporter 2 inhibitors, a more effective strategy should be explored to stop DN progression [80]. Based on the results of our study, maintaining intestinal microenvironment and promoting the dominance of butyrate-producing bacteria in gut may be a potential approach for DN amelioration. A few clinical trials have shown oral intake of probiotic supplementation conduces to renal function in patients with diabetes [81, 82], while fecal microbiota transplant is also an effective way [83]. To our knowledge, dietary education is another efficient method for patients with DN. Supplementation with SCFAs or adoption of a high-fiber diet or could mitigate renal inflammation [15, 60]. Currently, a few novel drugs targeting modulation of intestinal mucosal immunity showed a distinguished effect on glomerular disease treatment, which gave us prospects in the development of drugs targeting the gut-kidney axis for kidney disease treatment [84]. Qi et al. reported that microRNA-16 had the capacity to inhibit mesangial cells proliferation via toll-like receptor 4 signaling pathway in FCGR2B deficient mice [85]. Therefore, we conceive targeting the FCGR2B related signaling pathway, such as microRNA-16, might be a prospective therapy for DN and the future study is worthwhile to explore.

Our study owns a plenty of advantages. While a significant number of investigations have reported correlations and demonstrated variations in bacterial abundances in different cohorts, however, the specific reasons for these alterations and their causal relevance often remain uncertain. By using genetic variants, MR-Egger intercept and MR-PRESSO method, we prevented confounding factors and reverse causal effect to provide a robust causation between GM and DN. Besides, instrumental variations of GM were obtained from the latest comprehensive GWAS summary data which ensured the convincingness of MR results. We also performed a brand new-proposed multivariable MR methods (MR-BMA) to identify the most influential bacterial taxa on DN. Eventually, a PPI network was applied to understand the connection among GM related protein, while transcriptome MR analysis based on mapped genes supplied several potential biomarkers and therapeutic targets on DN.

Notably, this study also has some limitations: (i) The GWAS summary data from MibioGen only classified from phylum to genus and the causal effect of specific species belonged to each genus on DN could not be analyzed. (ii) The involved SNPs from MiBioGen and FinnGen dataset were derived from various cohort studies involved European. Thus, generalizing the MR results of this research to other racial populations might be not viable. (iii) The confounders recognized by Phenoscanner were removed according to the current studies and our clinical experience which indicated the potential bias. (iv) There were only 3283 DN cases in FinnGen cohort and such small sample size lacked great beta effect contributing to less statistical power. Nevertheless, this work was still worthy and provided an initial study using large scale genetic data to explore the correlation of GM and DN. Future study to include larger sample size from different database was expected. Eventually, we carried out a series of methods to validate the robustness of MR results and thus we thought our work was very valuable.

## Conclusions

In conclusion, we appraised the causality between gut microbiota and diabetic nephropathy through Mendelian Randomization analysis and demonstrated dysbiosis of GM increased risk of DN. Our study not only provides novel perspectives of GM on DN but opens avenues for potential strategies for DN precaution and therapy.

### Electronic supplementary material

Below is the link to the electronic supplementary material.

Additional file 1: Supplementary Table S1. MR analysis results of casual links between significant gut microbiota and DN. Table S2. Selected and proxy SNPs for each significant gut microbiota. Table S3. Reverse MR analysis of DN on GM. Table S4. Sensitivity analysis for reverse MR analysis. Table S5. Mapped gene by all significant bacterial taxa related SNPs. Table S6. Transcriptome MR results of causality of mapped genes and DN. Table S7. Heterogeneity and pleiotropy test results for transcriptomic analysis.

Additional file 2: Supplementary Figures

## Data Availability

The datasets supporting the conclusions of this article can be download online: gut microbiota GWAS data is derived from MibioGen (https://mibiogen.gcc.rug.nl/), diabetic nephropathy GWAS data is from FinnGen Release 5 (https://www.finngen.fi/en), mapped gene is from FUMA GWAS (https://fuma.ctglab.nl/), PPI data is from STRING(https://string-db.org/) and cis-eQTLs is from eQTLGen (https://eqtlgen.org/). The data generated or analyzed in this study are included within the article and its additional files.

## Abbreviations

GM: Gut microbiota
DN: Diabetic nephropathy
SCFAs: Short-chain fatty acids
GPR: G protein-coupled receptors
RAS: Renin-angiotensin system
MR: Mendelian Randomization
IVs: Instrumental variables
GWAS: Genome-wide association study
SNPs: Single nucleotide polymorphisms
IVW: Inverse variance weighted
MR-BMA: Mendelian randomization-Bayesian model averaging
PP: Posterior probability
MIP: Marginal inclusion probability
MACE: Model-averaged causal effect
eQTLs: Expression quantitative trait loci
PPI: Protein-protein interaction
FDR: False discovery rate
OR: Odds ratio
CI: Confidence interval
BC: Betweenness centrality
IL: Interleukin
T2DM: Type 2 diabetes mellitus
HDAC: Histone deacetylase
Syk: Spleen tyrosine kinase
