# Supplementary material for: Causal effect of gut microbiota and diabetic nephropathy: a Mendelian randomization study
Source: Diabetol Metab Syndr. 2024 Apr 24;16:89. doi: 10.1186/s13098-024-01327-7 (PMC11044463; doi:10.1186/s13098-024-01327-7)
Supplement: Supplementary file 2 — Additional file 2: Supplementary Figures [file 13098_2024_1327_MOESM2_ESM.docx]

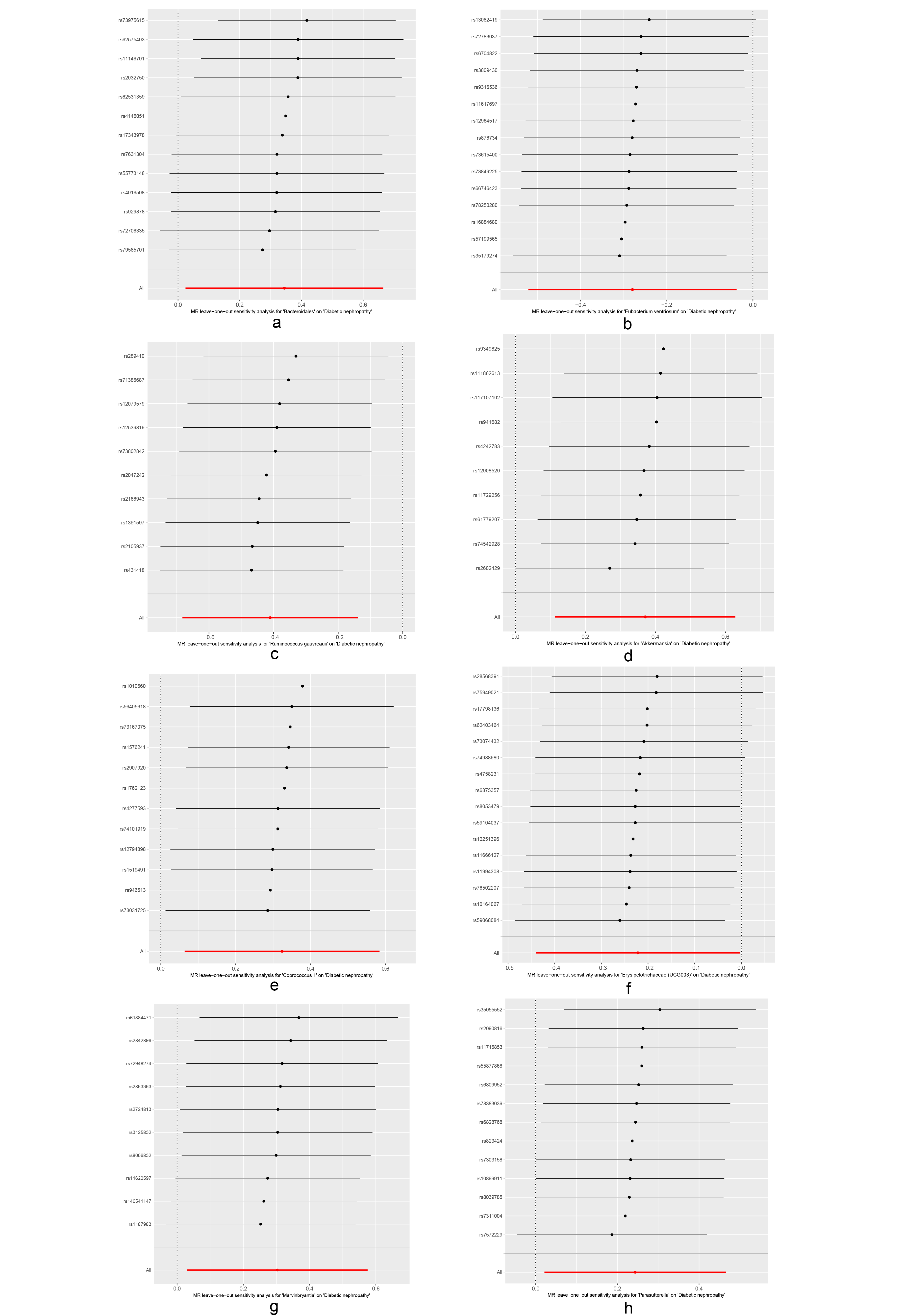


Figure S1. Leave-one-out sensitivity analysis for each significant bacterial taxon. Figure S1 a to h represent order Bacteroidales, genus Eubacterium ventriosum, genus Ruminococcus gauvreauii, genus Akkermansia, genus Coprococcus 1, genus Erysipelotrichaceae (UCG003), genus Marvinbryantia and genus Parasutterella, respectively. *MR, Mendelian Randomization.*


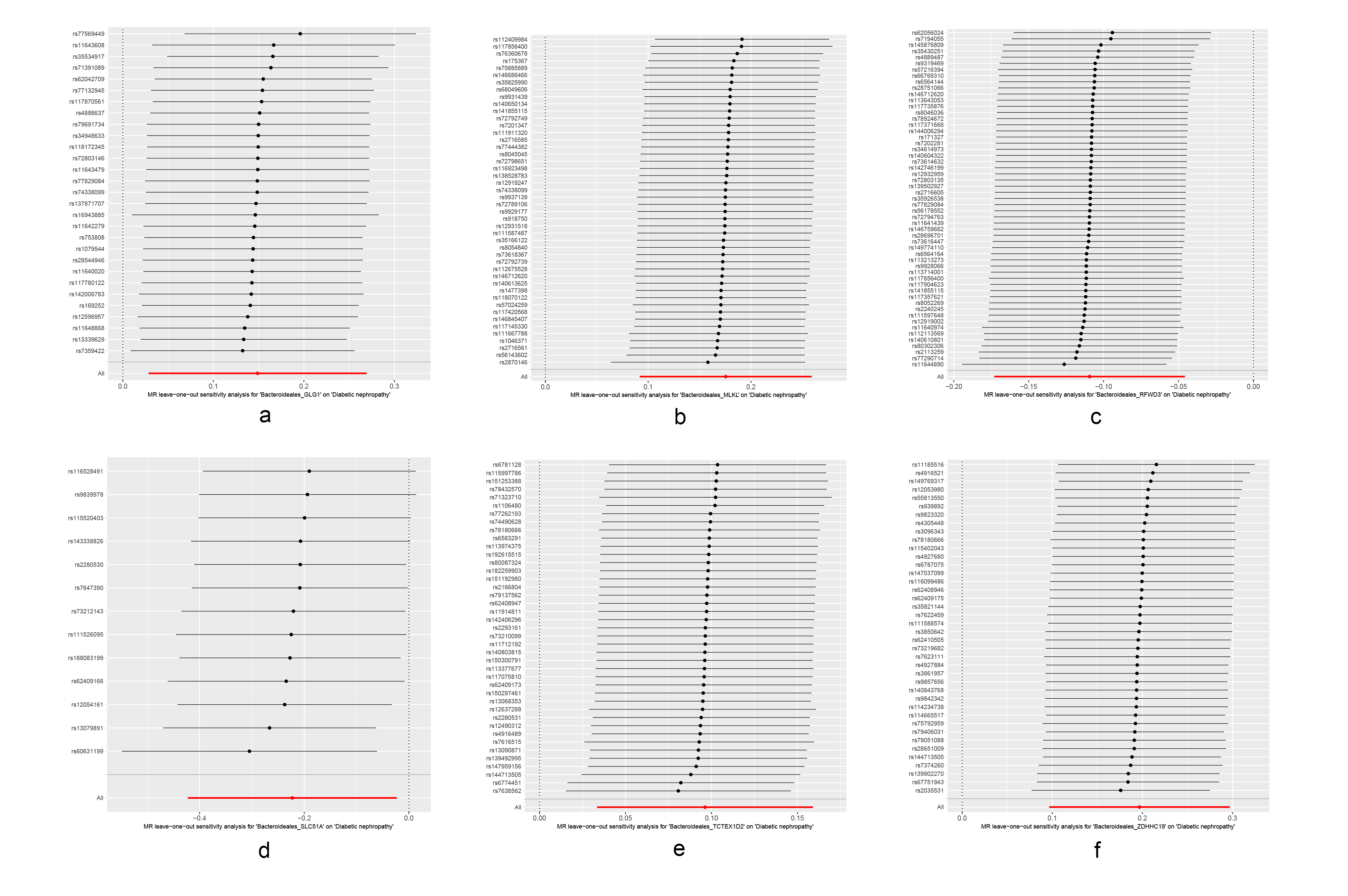


Figure S2. Results of Leave-one-out analysis for order Bacteroidales related mapped genes. Figure S2 a to f represent GLG1, MLKL, RFWD3, SLC51A, TCTEX1D2 and ZDHHC19, respectively. *MR, Mendelian Randomization.*


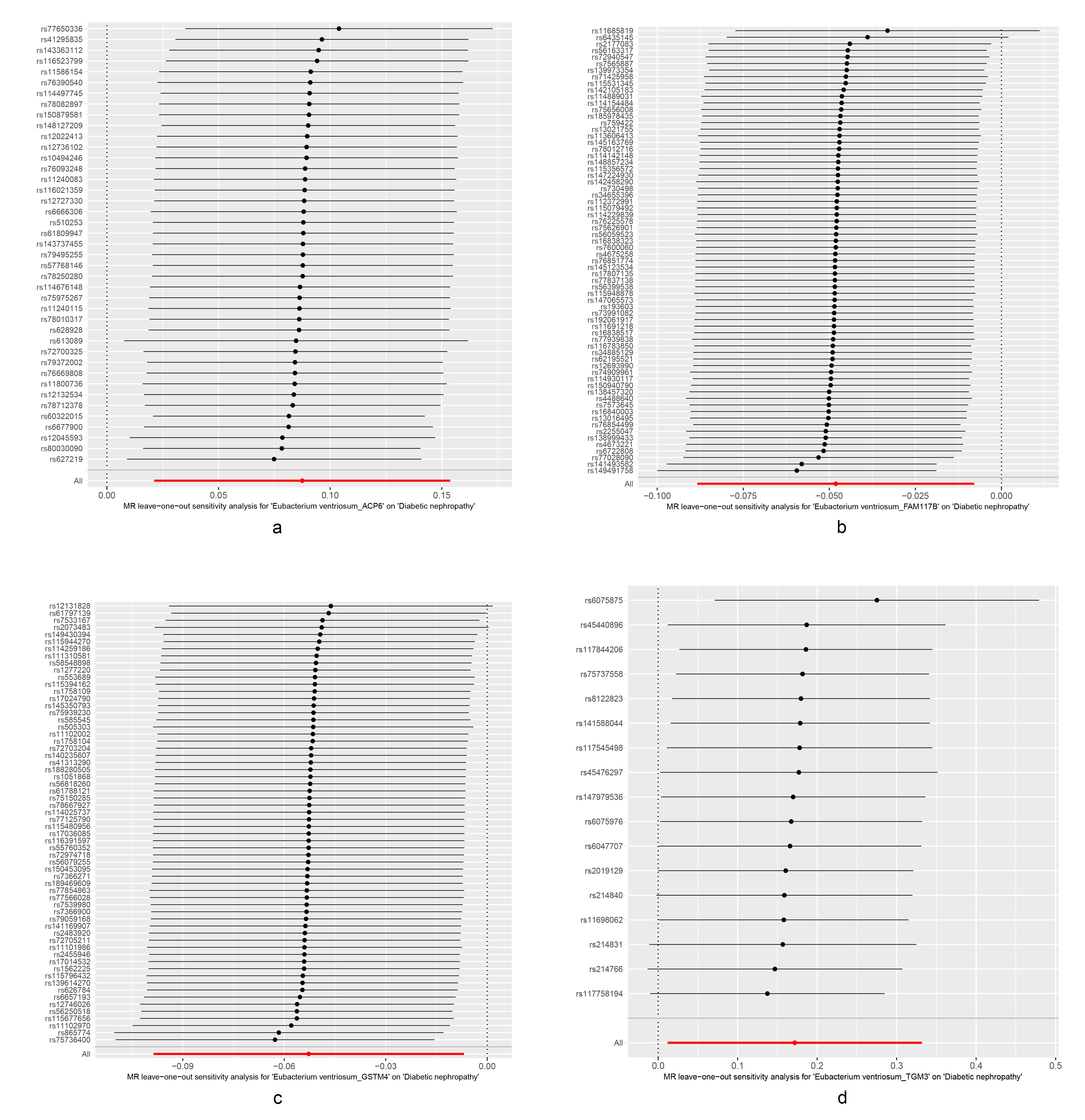


Figure S3. Results of Leave-one-out analysis for genus Eubacterium ventriosum related mapped genes. Figure S3 a to d represent ACP6, FAM117B, GSTM4 and TGM3, respectively. *MR, Mendelian Randomization.*


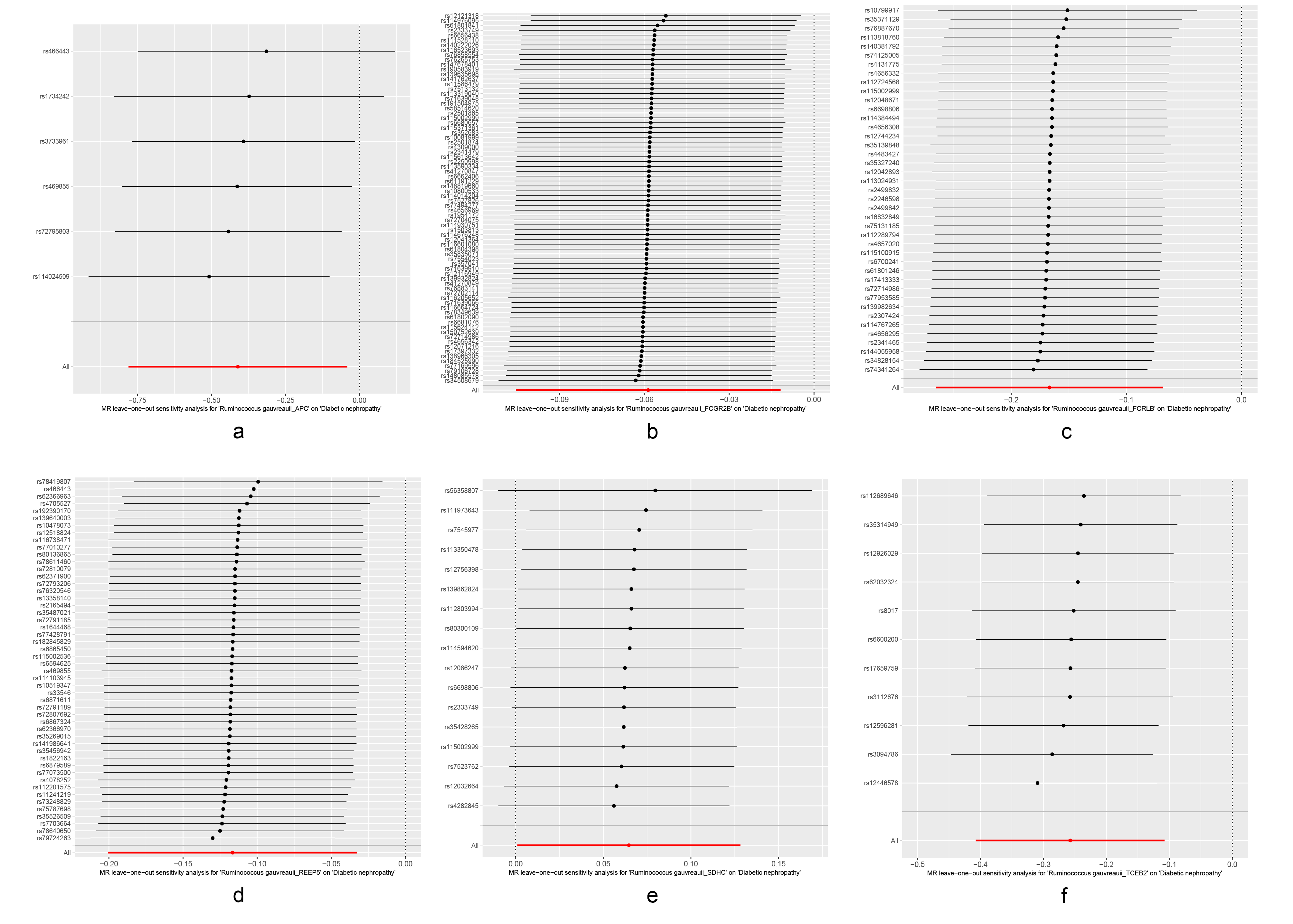


Figure S4. Results of Leave-one-out analysis for genus Ruminococcus gauvreauii related mapped genes. Figure S4 a to f represent APC, FCGR2B, FCRLB, REEP5, SDHC and TCEB2, respectively. *MR, Mendelian Randomization.*


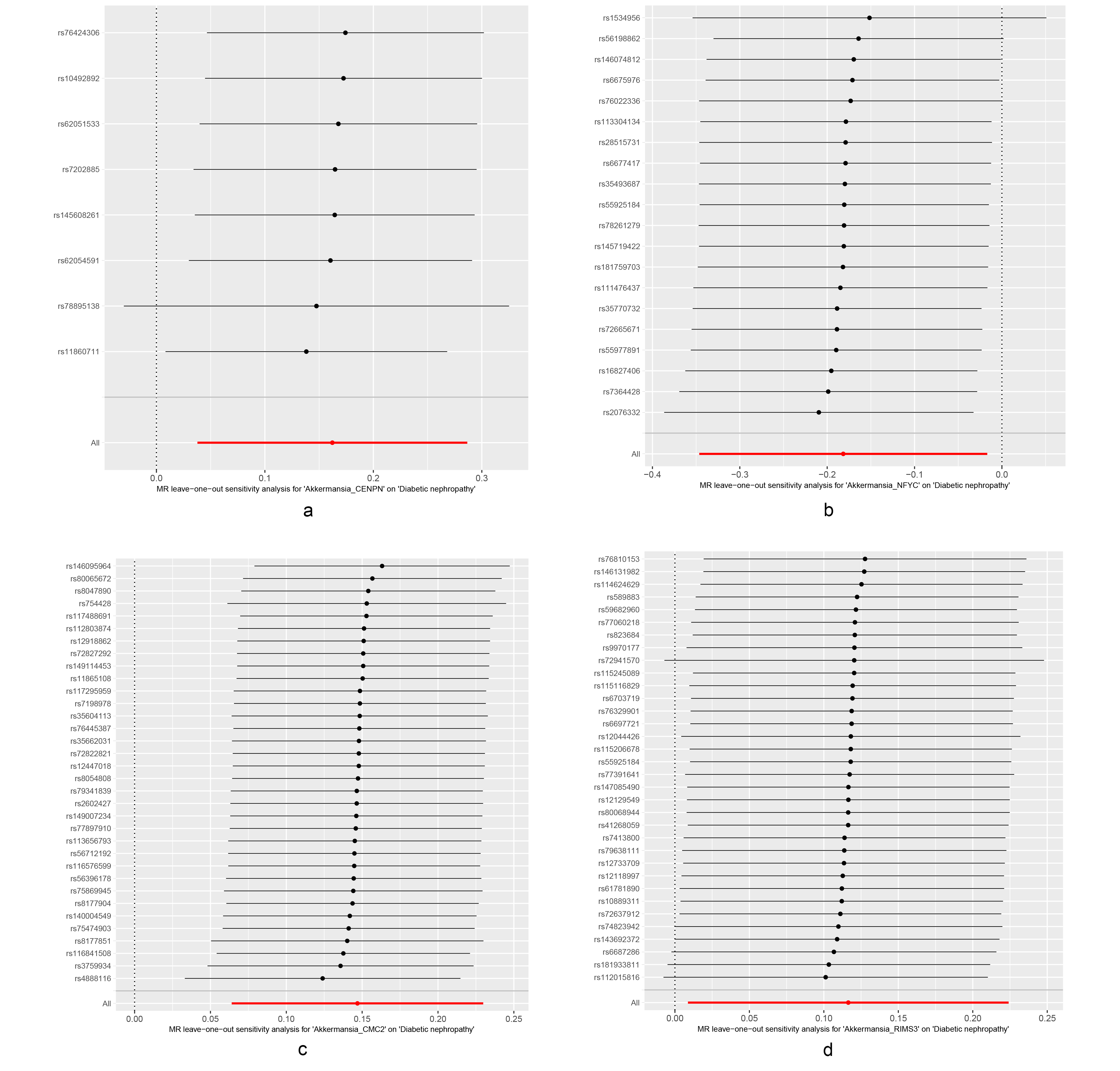


Figure S5. Results of Leave-one-out analysis for genus Akkermansia related mapped genes. Figure S5 a to d represent CEPNPN, CMC2, NFYC and RIMS3, respectively. *MR, Mendelian Randomization.*


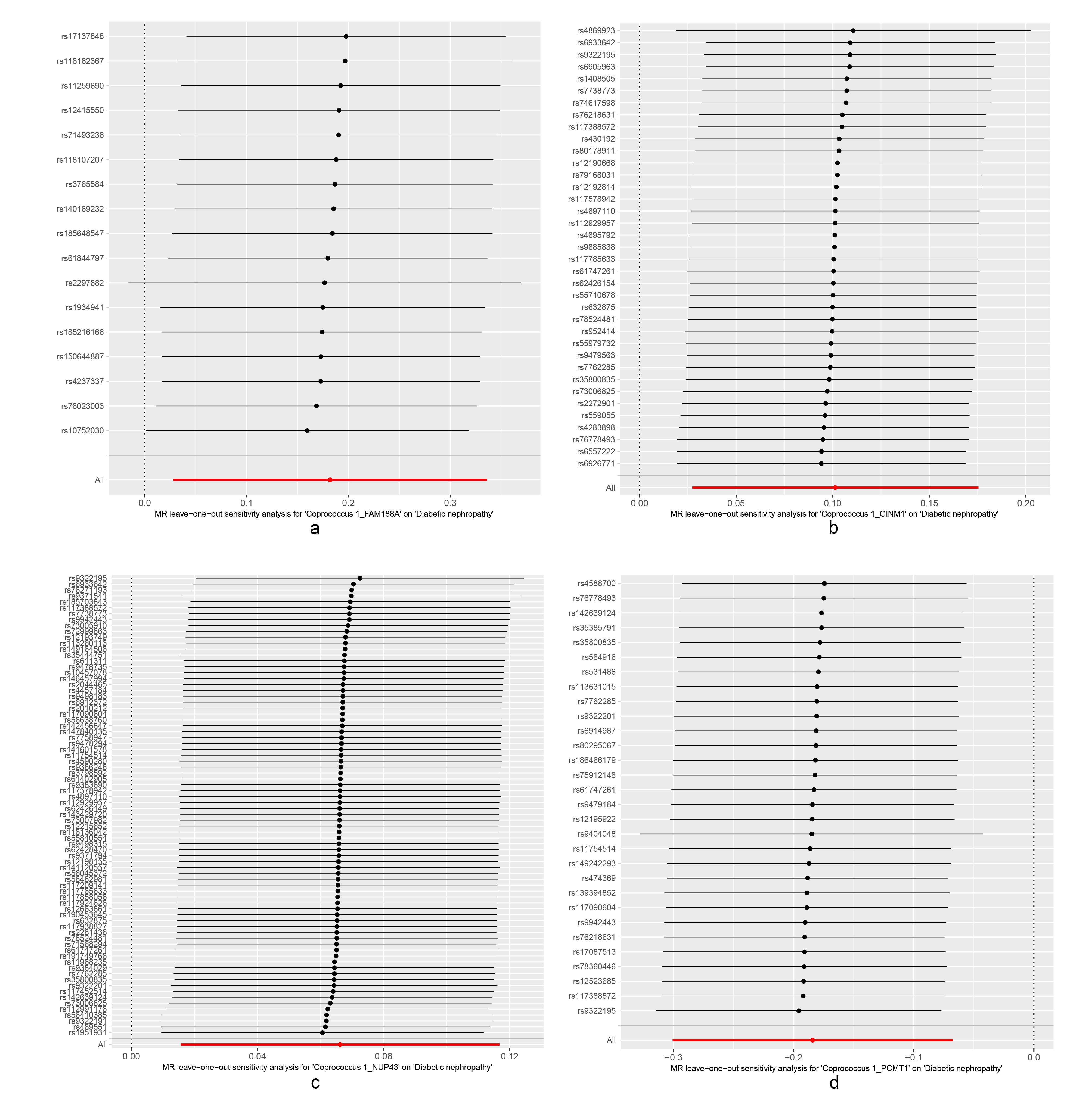


Figure S6. Results of Leave-one-out analysis for genus Coprococcus 1 related mapped genes. Figure S6 a to d represent FAM188A, GINM1, NUP43 and PCMT1, respectively. *MR, Mendelian Randomization.*


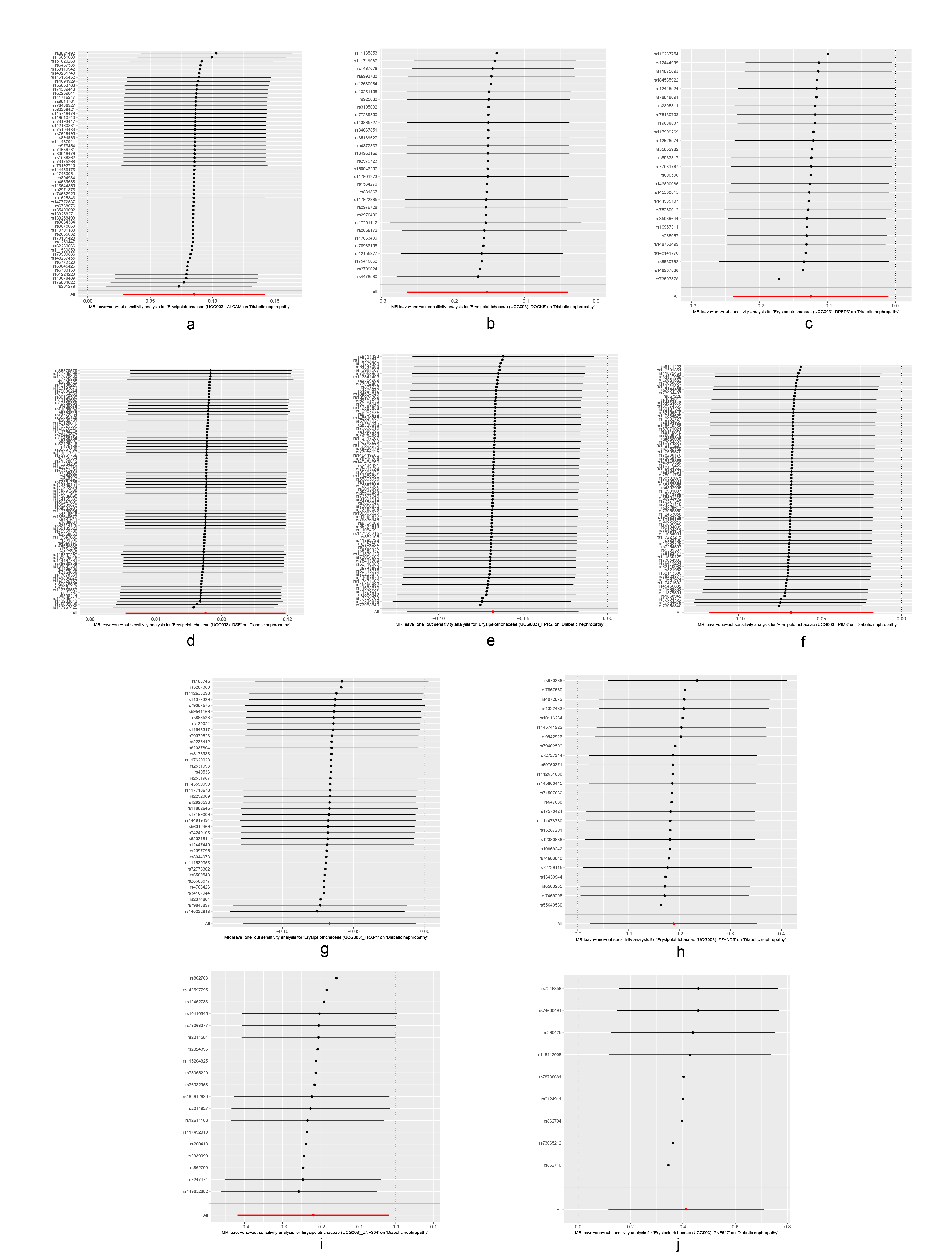


Figure S7. Results of Leave-one-out analysis for genus Erysipelotrichaceae (UCG003) related mapped genes. Figure S7 a to j represent ALCAM, DOCK5, DPEP3, DSE, FPR2, PIM3, TRAP1, ZFAND5, ZNF304 and ZNF547, respectively. *MR, Mendelian Randomization.*


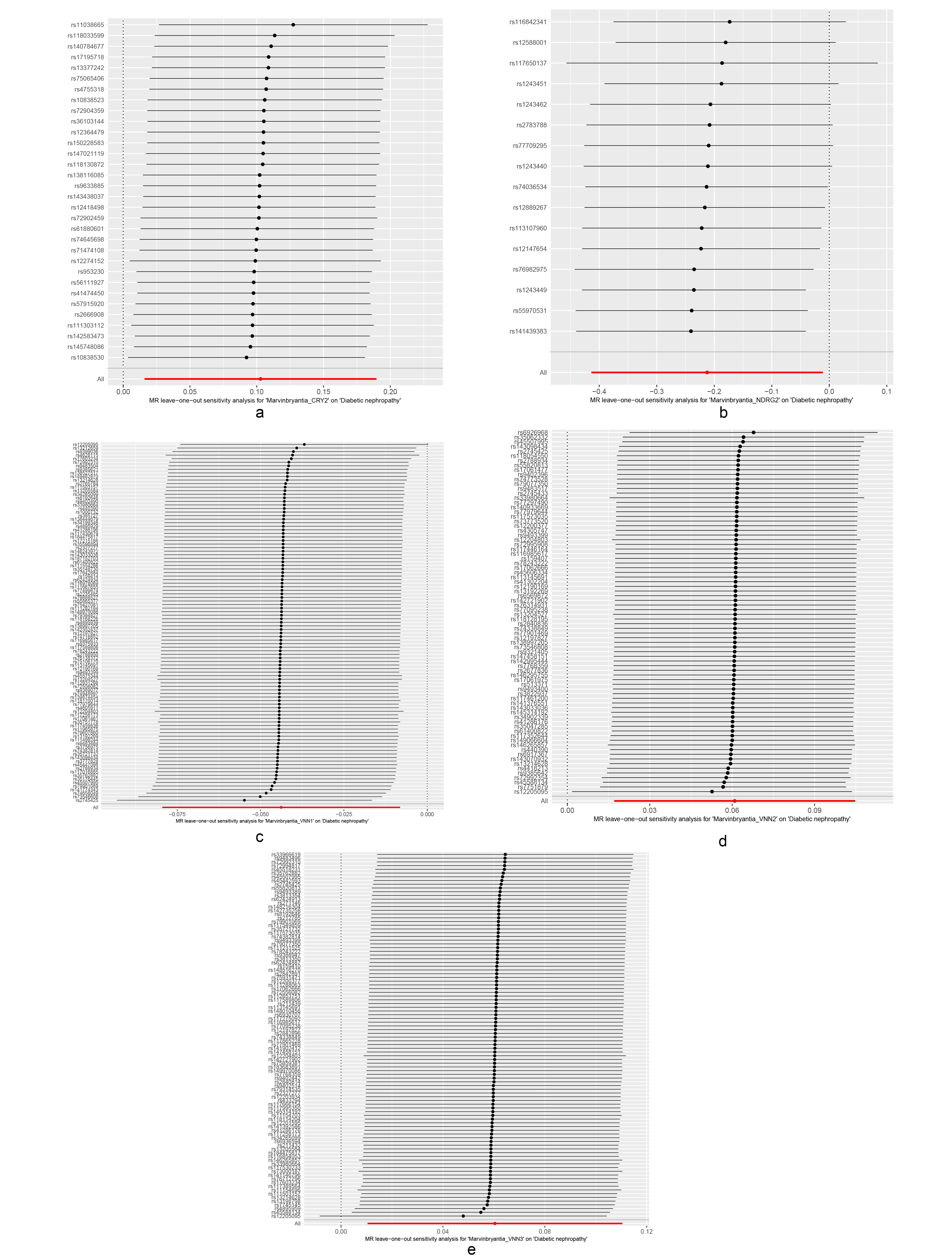


Figure S8. Results of Leave-one-out analysis for genus Marvinbryantia related mapped genes. Figure S8 a to e represent CRY2, NDRG2, VNN1, VNN2 and VNN3, respectively. *MR, Mendelian Randomization.*


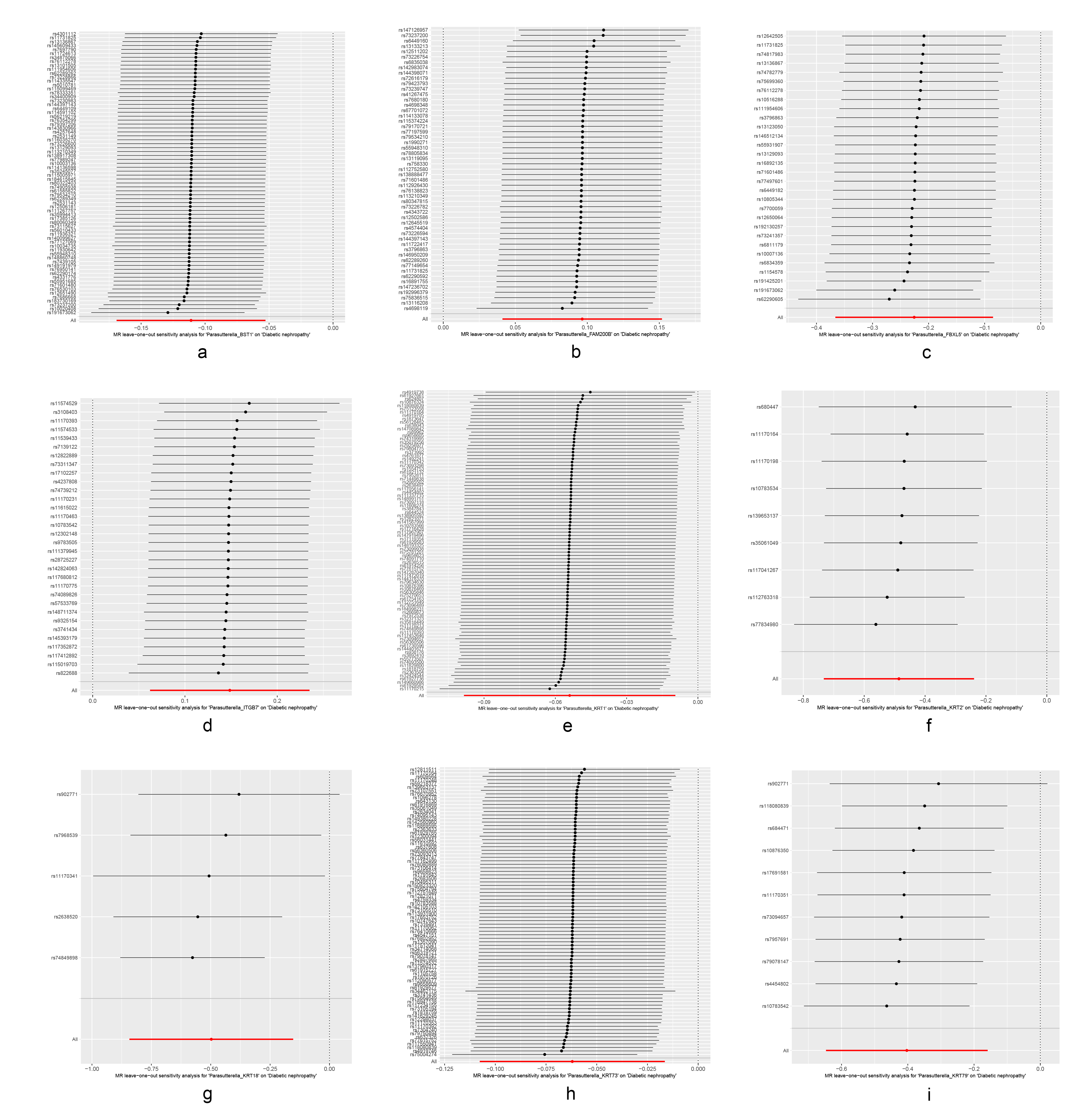


Figure S9. Results of Leave-one-out analysis for genus Parasutterella related mapped genes. Figure S9 a to i represent BST1, FAM200B, FBXL5, ITGB7, KRT1, KRT2, KRT18, KRT73 and KRT79, respectively. *MR, Mendelian Randomization.*
